# Characterization of Differentially Expressed Genes under Salt Stress in Olive

**DOI:** 10.3390/ijms23010154

**Published:** 2021-12-23

**Authors:** Soraya Mousavi, Roberto Mariotti, Maria Cristina Valeri, Luca Regni, Emanuele Lilli, Emidio Albertini, Primo Proietti, Daniela Businelli, Luciana Baldoni

**Affiliations:** 1 Institute of Biosciences and Bioresources, National Research Council, 06128 Perugia, Italy; roberto.mariotti@ibbr.cnr.it (R.M.); mariacristinavaleri.mcv@gmail.com (M.C.V.); emanuele.lilli96@gmail.com (E.L.); luciana.baldoni@ibbr.cnr.it (L.B.); 2 Department of Agricultural, Food and Environmental Sciences, University of Perugia, 06121 Perugia, Italy; luca.regni@unipg.it (L.R.); emidio.albertini@unipg.it (E.A.); primo.proietti@unipg.it (P.P.); daniela.businelli@unipg.it (D.B.)

**Keywords:** salt stress genes, *Olea europaea*, gene expression, climate change, salt tolerance, olive cultivars

## Abstract

Climate change, currently taking place worldwide and also in the Mediterranean area, is leading to a reduction in water availability and to groundwater salinization. Olive represents one of the most efficient tree crops to face these scenarios, thanks to its natural ability to tolerate moderate salinity and drought. In the present work, four olive cultivars (Koroneiki, Picual, Royal de Cazorla and Fadak86) were exposed to high salt stress conditions (200 mM of NaCl) in greenhouse, in order to evaluate their tolerance level and to identify key genes involved in salt stress response. Molecular and physiological parameters, as well as plant growth and leaves’ ions Na^+^ and K^+^ content were measured. Results of the physiological measurements showed Royal de Cazorla as the most tolerant cultivar, and Fadak86 and Picual as the most susceptible ones. Ten candidate genes were analyzed and their complete genomic, CDS and protein sequences were identified. The expression analysis of their transcripts through reverse transcriptase quantitative PCR (RT-qPCR) demonstrated that only *OeNHX7*, *OeP5CS*, *OeRD19A* and *OePetD* were upregulated in tolerant cultivars, thus suggesting their key role in the activation of a salt tolerance mechanism.

## 1. Introduction

The Mediterranean area is particularly sensitive to climate change [1], that will lead to a reduction in water availability and to groundwater salinization in the near future [2]. Olive tree (*Olea europaea* L.), considered one of the most important crops of the Mediterranean area, is able to face the environmental changes and develop adaptive mechanisms, thanks to its huge varietal patrimony [3,4,5,6]. Soil salinization is particularly menacing in the Mediterranean Basin, subject to water scarcity and high evapotranspiration demands, and olives are often irrigated with low-quality or wastewater, containing high concentrations of salts [7,8,9].

Although the olive tree is generally regarded as moderately tolerant to salinity, significant differences in salt tolerance have been reported among cultivars [7,10,11]. Several studies have focused on the response of different olive cultivars to salt stress [7,10,11,12,13], but the mechanisms involved in salt tolerance are still required to be adequately recognized.

Salt stress in plants can lead to ionic, osmotic and secondary stresses, particularly oxidative stress, that may inhibit leaf expansion, restrict photosynthesis and limit the accumulation of biomass [14]. In many plants, the adaptive response to salinity stress includes the active exclusion of sodium (Na^+^) ions and/or their sequestration into the vacuole, production of compatible solutes and detoxification of reactive oxygen species (ROS) [15]. In glycophytes, excessive Na^+^ often leads to K^+^ deficiency under salt stress [16], therefore, maintenance of a high K^+^/Na^+^ ratio may help plants to adapt to salt stress [16,17,18]. The olive plant has developed a series of mechanisms to tolerate and grow when prolonged soil salinization occurs [19]. These mechanisms include preventing salt translocation or decreasing its transport, excluding Na^+^ and Cl^−^ from leaves [9,20,21], or compartmentalizing toxic ions within leaves [22].

Several genes are involved in plant response to salt stress. *NHX7* is required for the maintenance of ionic homeostasis and, therefore, in the regulation of transport or compartmentalization of Na^+^ and/or K^+^ [23]. To counteract saline stress, Na^+^ may be sequestered in vacuoles [24] and Na^+^/H^+^ exchangers, controlled by *NHX* gene family, transport Na^+^ from the cytoplasm to the vacuole, a process driven by the H^+^ gradient established by vacuolar H^+^-pyrophosphatase and H^+^-ATPase [25]. The salt tolerant effect of *NHX* genes has been definitively established by the production of many salt-tolerant transgenic plants [26,27].

Phosphatidylinositol (PI) 4-kinases (*PI4Ks*) has been shown to confer salt stress tolerance in Arabidopsis plants [28] and its activity is also required for the induction of endocytic trafficking and lateral roots primordium formation [29].

The K^+^/Na^+^ ratio is also important for the growth rate of plants [30,31,32] and both low K^+^ and high Na^+^ can trigger cellular Ca^2+^ signaling, leading to the activation of complex molecular networks involved in plant growth [33]. Therefore, an important role in plant response to salt stress is played by Calcineurin B-like proteins (*CBLs*) [34].

In order to counter the negative effects of high salinity, another useful mechanism is regulating water/osmotic homeostasis through aquaporins (AQP) [35]. In plants, the MIP family is divided into five subfamilies, including the plasma membrane intrinsic proteins (PIPs) [36]. PIPs-mediated transcellular transport of water plays an important role in the maintenance of water homeostasis in plants under stress conditions [37,38,39].

The cysteine protease gene *RD19A* (Responsive to Dehydration 19A) was induced in Arabidopsis thaliana by water deficit and was responsive to salt stress [40]. The upregulation of this gene in tobacco transgenic plants enhanced their tolerance to salt stress [41], moreover, the expression of *RD19A* under 500 mM of NaCl in *Arabidopsis pumila* confirmed its significant upregulation under salt stress conditions [42]. Stress-related proteins (SRPs) are known to be involved in the promotion of stress tolerance and play a positive role in the biogenesis of lipid droplets [43,44,45]. In Arabidopsis, *SRP1* and *SRP3* were induced by high salinity [45].

Several studies have demonstrated that over-expression of the *P5CS* gene increases proline production and confers salt tolerance in transgenic plants. Proline increases cellular osmolarity (turgor pressure) that provides the turgor necessary for cell expansion under stress conditions and is a dominant organic molecule that accumulates in many organisms following exposure to environmental stresses, and especially under salt-stress conditions [46].

The family of the B-box (*BBX*) proteins [47] includes a class of zinc-finger transcription factors involved in regulatory networks and responses to abiotic stresses [48]. *BBX19* interacted with ABF3 to negatively regulate drought tolerance in *Chrysanthemum morifolium* [49]. *BBXs* enhanced salt tolerance in Arabidopsis compared to wild-type plants [50]. In chloroplast genome, *PetD* (CytB6) is a component of the plastoquinone-plastocyanin reductase [51], involved in electron transport and generation of ATP. Mousavi et al. [10] reported that *OePetD* was increasingly upregulated in the susceptible olive cultivar Koroneiki, while, in the tolerant cultivar Royal de Cazorla, the expression of *OePetD* did not show significant variations under stress conditions.

Although there are several studies on genes involved in salt stress response in different plant species, very few ones have reported on salt inducible genes in olive. Some transcription factors were highly regulated under salt stress in tolerant and susceptible olive cultivars [52] and salt-responsive transcripts were identified [53], some genes were differentially methylated under salt stress [10], meanwhile Poku et al. [54] have demonstrated that the olive *OeSRC1* gene may increase salt and drought tolerance in tobacco plants, and an extensive EST-SNP genotyping of numerous olive cultivars and wild plants has allowed identification of salt-stress-responsive homologous genes in olive [55]. The selection of genes to include in this study was based on the following criteria: (i) genes involved in salt stress response mechanisms in other plant species, such as *NHX7*, *NHX6*, *P5CS* and *BBX19*; (ii) genes already found differentially expressed in olive under salt stress [10], as *PIK4g*, *PIP1.1* and *PetD* and (iii) some genes derived from transcripts related to the mechanisms of tolerance to saline stress in wild and cultivated olives [55] *CBL3*, *RD19A* and *SRP.*

The present study aims to characterize the tolerance level of some olive cultivars to high salinity stress and to explore differential regulation of some genes putatively involved in salt stress response in olive, through their expression profiling on cultivars under saline stress.

## 2. Results

### 2.1. Plant Growth and Visible Symptoms

**Figure 1:**
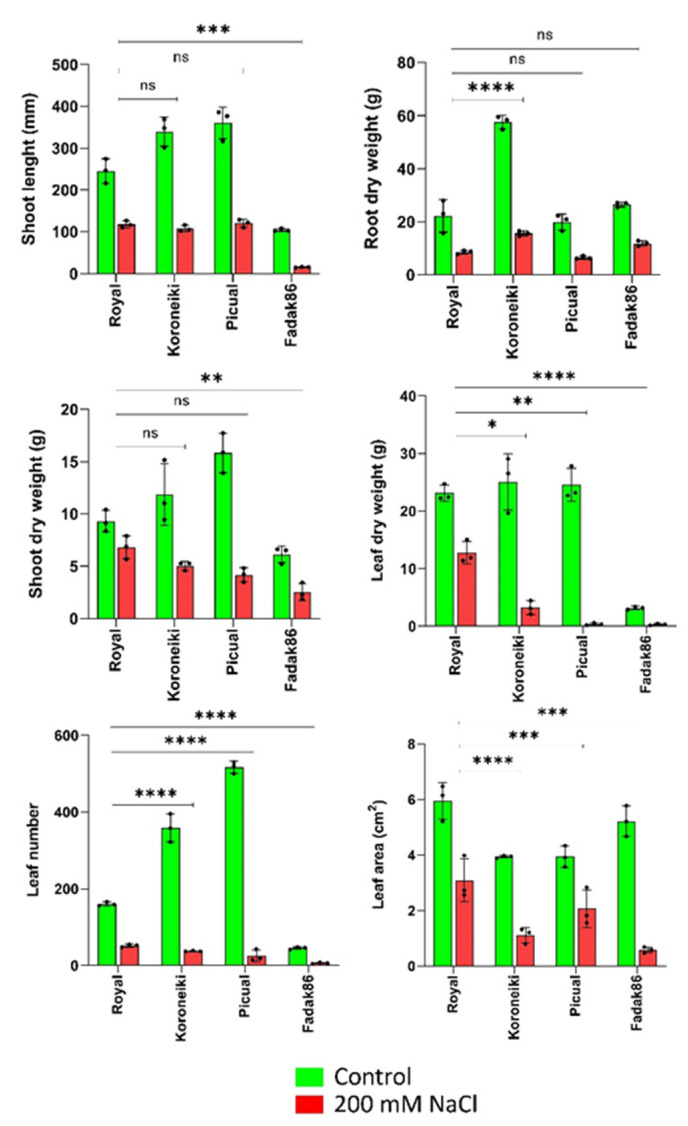
Plant growth measurements of four cultivars at 0 (green) and 200 mM NaCl (red), after 240 DATS of salt stress. Bars represent means ±SD of three replicates. Asterisks show **** *p* = 0.000, *** *p* < 0.001, ** *p* < 0.01 and * *p* < 0.05 and ns represents not significant values.

Salt treatments reduced dry weight in all plant organs of examined cultivars at 200 mM NaCl, but higher reductions were observed in leaves (Figure 1). Shoot length and leaf area in cv. Fadak86 under salt stress decreased by 85.40 and 88.90%, respectively, while in cv. Picual leaf number, and their dry weight, faced the highest reduction (95 and 98.47%, respectively).

Growth reduction in the Royal de Cazorla (Royal) cultivar was the lowest for almost all measured plant organs (Figure 1).

**Figure 2:**
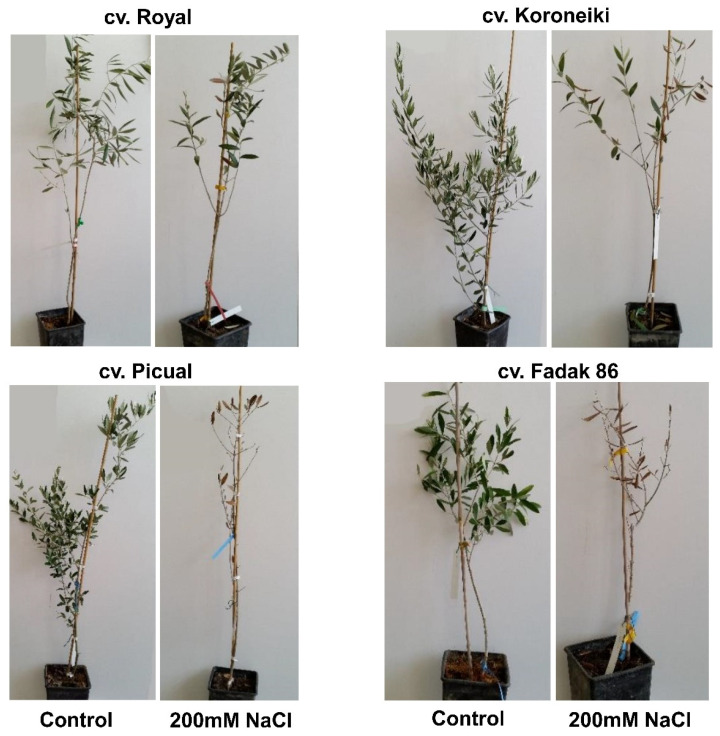
Plants after 240 DATS under control and salt stress conditions (200 mM of NaCl) of cvs. Royal, Koroneiki, Picual and Fadak86.

Other remarkable differences among the studied cultivars were observed at the end of the experiment (240 days after treatment start, DATS) (Figure 2). Fadak86 plants faced the most severe stress, with a low number of green leaves and a reduced plant growth. A similar feature was observed for cv. Picual. In fact, visible symptoms of stress were observed also at 180 DATS for these two cultivars. On the contrary, in Royal and Koroneiki plants, only some brown leaves were observed at the end of experiment, even if, in cv. Koroneiki, the fallen leaves were more than cv. Royal. In tolerant cultivar Royal, no plant died, but at the same time did not show visible bud, leaf and shoot growth under salt conditions (Figure 2).

### 2.2. Mineral Leaf Content

**Figure 3:**
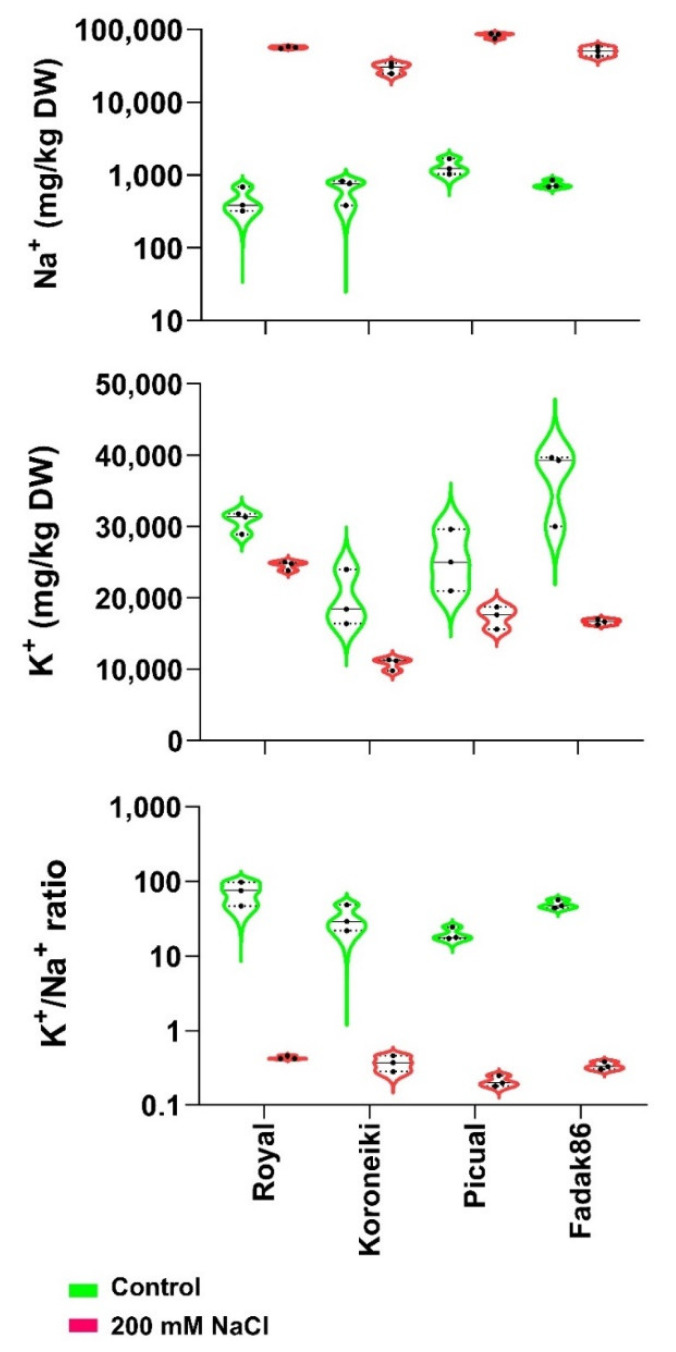
The violin graphs for Na^+^ and K^+^ ions and their ratio in leaves of analyzed plants. Each plot shows the distribution of data for four cultivars from the minimum to the maximum level, with horizontal inner lines showing the median. Dot lines represent the lower and upper limits of the first and third quartiles. The horizontal width of the violin depends on data density.

The Na^+^ ion content measured on control and treated plants at 240 DATS, strongly increased under salt stress in all studied cultivars (Figure 3). The highest concentration of Na^+^ was measured in cv. Picual and the lowest one in cv. Koroneiki. On the contrary, K^+^ concentration decreased in all salt treated plants with respect to the control ones. The lowest K^+^ ion reduction under 200 mM of NaCl was observed in the leaves of Royal cultivar, and the highest decrease with respect to control was revealed in cv. Fadak86. The highest K^+^/Na^+^ ratio under stress condition was shown by cv. Royal and the lowest one was measured in cv. Picual.

### 2.3. Identification and Structural Analyses of Selected Genes

Homologous mRNA and CDS of ten most salt-stress-responsive genes were used as query against cvs. Farga, Leccino, Picual and var. *sylvestris* genomic sequences. Finally, genomic, CDS and protein sequences were fully or partially obtained for all four genomes (Table 1 and Table 2).

**Figure 4:**
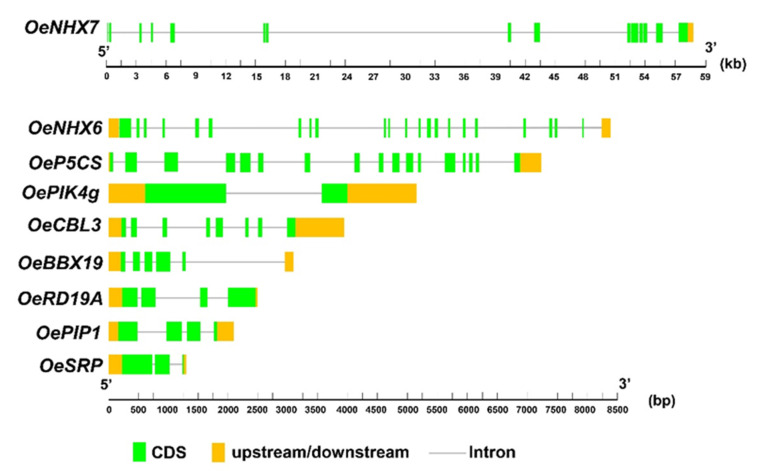
Gene structure of ten genes was identified in the present study in olive cvs.

*OeNHX7* Na^+^/H^+^ and K^+^/H^+^ antiporter gene, with a length of 58,522 bp, was the longest gene among the studied ones. This gene was characterized by 16 exons and 15 introns including a CDS (2646 bp), coding for 882 amino acids (aa). The complete protein sequence predicted in cv. Farga genome was the same as published for var. *sylvestris* (XP_022891851.1) without any amino acid change. The *OeNHX7* gene in *sylvestris* genome was placed on chromosome 12, while in cv. Leccino it was placed in linkage group 6. This gene was completely predicted in all four genomes, but the first part was missing in the CDS (Oleur061Scf1390g01001.1) of cv. Picual (Table 1 and Table 2, Figure 4).

*OeNHX6* Na^+^/H^+^ and K^+^/H^+^ antiporter gene, was fully predicted in cv. Farga genome, with a total length of 7410 bp. This gene was reported in chromosome 13 of var. *sylvestris* and linkage group 8 of cv. Leccino and was characterized by 22 exons and 22 introns. The complete protein sequence was found in three genomes, except that of cv. Leccino, with a total length of 524 amino acids in cv. Farga and var. *sylvestris*, and 523 aa in cv. Picual, that also showed 26 changes in aa sequence when compared with the sequences of the other cultivars here analyzed (Table 1 and Table 2, Figure 4 and Figure S1). The regulatory elements (REs) detection showed five and six different REs in cv. Farga and var. *sylvestris* in the upstream part, with five and four REs in the downstream part of the same cultivars, respectively (Table S1). Cultivars Leccino and Picual were not analyzed since the gene was not complete (Table 1).

*OeP5CS* gene, involved in proline biosynthetic process, with a total length of 6877 bp, was completely predicted in all four genomes. *OeP5CS* gene is located in chromosome 13 of var. *sylvestris* and linkage group 8 of cv. Leccino. This gene included 17 exons and 16 introns. The protein sequences were composed by 631 aa in all genomes. The published CDS (Oleur061Scf2321g01015.1) of cv. Picual was different in length and in nucleotide composition with respect to what was predicted in the present study. Picual cultivar had one amino acid change at position 267 in which methionine was replaced by isoleucine, while var. *sylvestris* had two amino acid changes at position 45 and 267. In cv. Farga, five amino acid changes were found (Table 1 and Table 2, Figure 4 and Figure S1). No polymorphisms were detected in the upstream/downstream part of the gene in each analyzed genome.

*OePIK4g* gene, involved in cellular response to hypoxia, with a total length of 5122, was predicted in cvs. Farga and Picual and previously published in cv. Leccino (MF958940.1). The published *OePIK4g* in var. *sylvestris* was not complete (XM_023040706.1). This gene was located in chromosomes 15 in var. *sylvestris* and in linkage group 23 of cv. Leccino, and was characterized by two exons and one intron. The protein sequences were 581 aa long in all three genomes, with only one amino acid change in position 166 in cv. Farga, where a histidine was replaced by an arginine (Table 1 and Table 2, Figure 4 and Figure S1). The analysis of RE in upstream/downstream part of polymorphic genomes showed 29, 26 and 30 REs in cvs. Farga, Leccino and Picual, respectively. Var. *sylvestris* was not analyzed since the gene sequence was not complete (Table 1 and Table S1).

*O*gene, a calcium ion binding with 3887 bp length, was completely predicted in Farga and Picual cultivars. This gene was characterized by eight exons and seven introns. *OeCBL3* placed in chromosome 7 of var. *sylvestris* and in linkage group 8 of cv. Leccino. The complete protein sequence was composed by 224 amino acids, without any change in the above-mentioned genomes (Table 1 and Table 2, Figure 4). There was not any polymorphism in the upstream/downstream part of the gene in cvs. Farga and Picual.

*OeBBX19* gene, with a zinc ion binding molecular function, had a total length of 3175 bp and was completely predicted in the genomes of Farga and Leccino cultivars. This gene is characterized by five exons and five introns. The published *OeBBX19* gene in var. *sylvestris* (XP_022876209.1) was similar to the predicted ones, while in cv. Picual was not complete. This gene is located in chromosomes 5 of var. *sylvestris* and in linkage group 23 of cv. Leccino, respectively. The protein sequence was 216 aa long in all three genomes without any amino acid changes (Table 1 and Table 2, Figure 4). RE detection was performed in cvs. Farga and Leccino, since there was not any polymorphism among the latter one and var. *sylvestris*. In the upstream part, six and 10 REs were found in cvs. Farga and Leccino, respectively, and the same number of REs were detected in the downstream part (Table S1).

*OeRD19A* gene, with a cysteine-type endopeptidase activity, had a total length of 2471 bp and was completely predicted in cvs. Farga and Leccino. This gene is placed in linkage group 6 of cv. Leccino and characterized by four exons and three introns. The complete protein sequence is composed by 377 aa in three genomes except cv. Picual, without any amino acid change among the genomes (Table 1 and Table 2, Figure 4). Only for cvs. Farga and Leccino was it possible to predict the complete gene and then RE analysis was performed in these genomes. The upstream part did not show any polymorphism among the two cultivars, while the polymorphic downstream showed two REs in cv. Farga and three in cv. Leccino (Table S1).

*OePIP1.1* gene, with a water channel activity and total length of 2109 bp, was completely predicted in cvs. Farga and Leccino and sequence information is also reported for two other published genomes (cv. Picual and var. *sylvestris*) (Table 1). This gene was located in chromosomes 19 of var. *sylvestris* and in linkage group 14 in cv. Leccino and identified by four exons and three introns. The protein sequence length was 285 aa in all four genomes, with an amino acid change in position 85 of cv. Leccino where lysine was replaced by glutamine (Table 1 and Table 2, Figure 4 and Figure S1). RE analysis was performed in the upstream part of the gene in cvs. Farga, Leccino and Picual, var. *sylvestris* had the same sequence as cv. Farga in both UTRs. Five, six and five REs were detected in cvs. Farga, Leccino and Picual, respectively. In the downstream part, cv. Farga had the same sequences of var. *sylvestris* and cv. Picual with five REs, while in cv. Leccino seven REs were detected (Table S1).

For *OeSRP* gene there was no molecular function reported in Uniprot, while it seemed involved in several biological processes. Its nucleotide sequence showed a total length of 1347 bp, and it was completely predicted in all four genomes, with three exons and two introns. *OeSRP* gene was located in chromosome 20 of var. *sylvestris* and in linkage group 17 of cv. Leccino. The complete protein is composed by 250 aa; the only difference between the four genomes resides in position 107 and 236, where in the first one a glycine is present in cvs. Leccino and Picual and glutamine in cv. Farga and var. *sylvestris* and aspartic acid in the latter position (Table 1 and Table 2, Figure 4 and Figure S1). In the upstream part cv. Leccino showed the same sequence as cv. Picual, while cv. Farga was the same as var. *sylvestris*. The RE analysis showed seven and six regulatory elements in cvs. Farga and Leccino, respectively. In four analyzed genomes the downstream part did not show any polymorphism (Table S1).

Finally, the chloroplast gene *OePetD*, with an electron transporter role, had the total length of 160 bp without any polymorphism among the four protein sequences (Table 1 and Table 2, Figure 4).

### 2.4. Gene Expression Analysis

**Figure 5:**
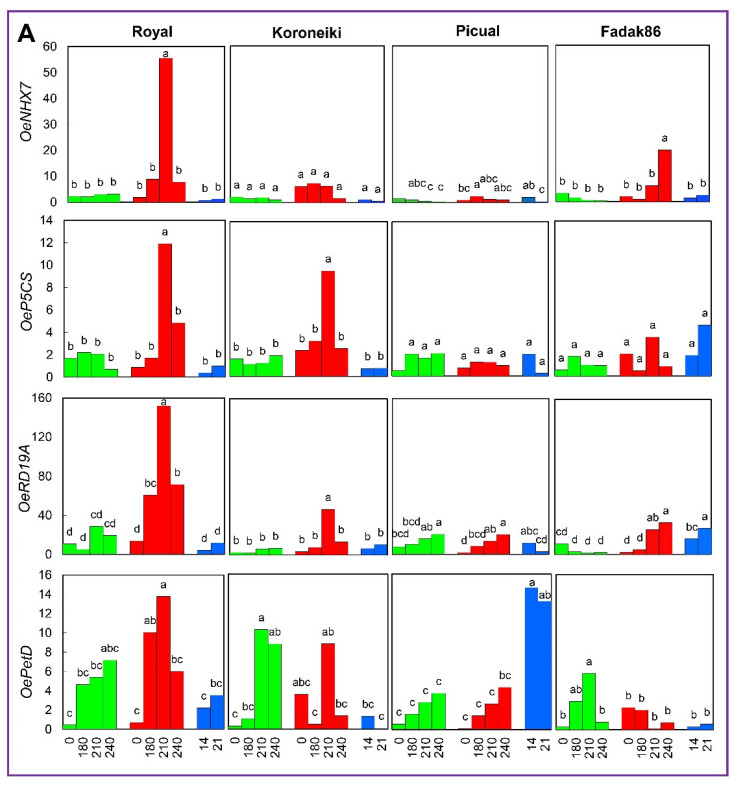

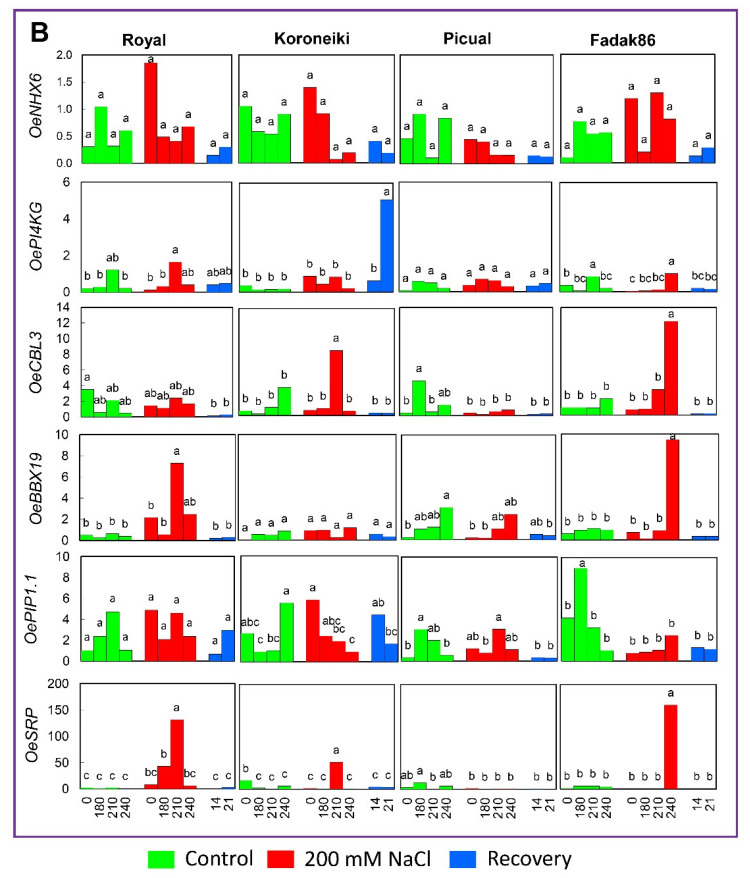
Gene expression under salt stress, as determined by RT-qPCR. (**A**) Genes highly expressed in tolerant olive cultivars. (**B**) differentially expressed genes in tolerant and susceptible cultivars. Leaves of plants under control conditions or treated at 200 mM NaCl were analyzed at time point 0, 180, 210 and 240 DATS. Plants in recovery at 14 and 21 DARS. Values are means of three biological replicates and three technical replicates. Different letters correspond to significantly different values at *p* ≤ 0.01.

The RT-qPCR experiments showed the expression profiles of the 10 selected genes at 0, 180, 210, 240 DATS and 14 and 21 days after recovery start, DARS (Figure 5).

A sharp increase of *OeNHX7* expression was observed in Royal under salt treatment at 210 DATS, about 50 folds with respect to control plants at the same time point, followed by a decrease to the last sampling date. A significant increase was also obtained after 240 DATS in Fadak86 treated plants, 10 folds with respect to the control plants at the same time. In cvs. Picual and Koroneiki the expression levels were very low in all cases. The expression of this gene showed very low regulation in control plants, as well as during the recovery from stress (Figure 5A).

The expression of *OeP5CS* in Royal and Koroneiki plants under stress showed a significant upregulation of about six and five folds, respectively, compared to control plants at 210 DATS. No significant variations have been noticed for cvs. Picual and Fadak86 at all treatments and sampling times (Figure 5A).

A high and significant increase in the relative abundance of *OeRD19A* gene transcripts was observed in the salt-tolerant Royal cultivar under stress, with a peak reached at 210 DATS, four folds compared to the control plants at the same time. All other cultivars showed similar but lower patterns of increase under the same conditions. The expression of this gene remained low also in control plants and in plants under recovery (Figure 5A).

*OePetD* had a slight increase in expression at experiment start (when salt concentration reached 200 mM) in Fadak86 with respect to control plants at the same time and then the expression decreased along the time (Figure 5A).

In Picual cultivar the pattern of expression was the same in control and stressed plants, a slight increase was observed in plants under salt conditions at 240 DATS. Indeed, in plants under recovery conditions, the expression had significant increase at 14 DARS and then had a slight decrease at 21 DARS. *OePetD* had the variation in expression in the Koroneiki cultivar with respect to the control plants at the same time points, anyway upregulation was at the beginning in stressed plants and then decreased during the time. In salt-tolerant Royal plants, *OePetD* expression increased in both control and treated plants, reaching a plateau at 240 and 210 DATS, respectively. In cv. Picual the expression levels were not so different, excluding plants under recovery. In Fadak86 cultivar the expression decreased during the time in stressed plants (Figure 5A).

No significant variations were found in the expression levels of *OeNHX6* gene for all treatments and all tested cultivars (Figure 5B).

In relation to *OePI4Kg4*, only Royal and Fadak86 plants under salt treatment experienced a significantly high expression of this gene at 210 and 240 DATS, respectively. Only low and not significant expressions were observed in Picual cultivar at all treatments and time points. *OePI4Kg4* showed the highest expression in plants of Koroneiki cultivar after 21 days of recovery (Figure 5B).

*OeCBL3* showed a progressive increase of expression in Fadak86 plants, five folds of increment with respect to control plants up to 240 DATS. A similar increase was found in Koroneiki at 210 DATS, an enhancement that was lost at 240 DATS. The other two cultivars did not show significant expression under salt stress with respect to control ones (Figure 5B).

The expression of the B-Box zinc finger (*OeBBX19*) gene, increased after 210 DAT in stressed plants of Royal and after 240 DATS in Fadak86, four and five folds, respectively. The expression in stressed plants of Picual was progressively increased with respect to control plants, but the increment was not considerable, indeed, in Koroneiki cultivar, the expression was always low with no significant differences (Figure 5B).

The expression of *OePIP1.1* was significantly higher in control plants of Fadak86 than in plants under salt stress, with a peak at 180 DATS followed by a continuous decrease. In salt-tolerant Royal plants, the gene was not significantly regulated under different conditions. In Koroneiki plants, *OePIP1.1* expression decreased progressively in plants under salt stress, and in Picual it reached a peak at 210 DATS, but similar levels were reached also in control plants at an earlier time point. Also, at 14 days after recovery, the expression was high and then decreased at 21 days (Figure 5B).

The *OeSRP* gene underwent an increase in expression 150-, 50- and 200-folds with respect to control plants in Royal, Koroneiki and susceptible Fadak86 plants after 210, 210 and 240 DATS, respectively. In Picual cultivar *OeSRP* did not show any upregulation under all treatments and stages (Figure 5B).

## 3. Discussion

Climate change, taking place also in the Mediterranean area, can lead to a reduction in water availability, an increase in temperatures and the need to use salty lands and water. Olive growing has expanded in recent decades outside the borders of the Mediterranean, reaching different continents, often affected by scarcity of water and nutritional resources and by phenomena of salinity of soils and irrigation water [57,58].

Olive represents one of the most efficient crops to face these scenarios, thanks to its natural ability to tolerate aridity and produce under limited environmental resources, but the challenges posed by the new climate constraints impose the need to explore the high variability of the species in relation to the abiotic stress tolerance and to characterize the genetic determinants controlling plant response to salt stress.

These goals can be achieved more rapidly thanks to the new genomic tools under development for olive, accelerating the discovery of candidate genes linked to abiotic tolerance. Differentially expressed genes related to salt tolerance were identified in olive [52,53], showing that tolerance to high salinity depends on the genotype, as demonstrated by a lot of other evidence [10,11,22,59], and that this trait is associated with the ability of olive trees to retain Na^+^ and Cl^−^ ions in the roots [9,13,60].

Salinity induces changes in gene expression at global scale. Salt tolerance in olive cultivars is associated with effective mechanisms of ion exclusion and retention of Na^+^ and Cl^−^ in the root, limiting the accumulation of these ions into actively growing shoots. It is more likely that K^+^/Na^+^ exchange at the plasmalemma is involved in regulating the transport of Na^+^ to the shoot by preventing apoplastic transport into the xylem [59].

In the present study, plant growth parameters and visible symptoms have been recorded in four olive cultivars under 200 mM of NaCl. Among the studied cultivars, the most susceptible ones were Fadak86 and Picual, that showed a harsh decrease in growth and, by increasing stress duration, plants have faced the noxious concentration of NaCl, leading to the death of several replicates. In the same condition, Koroneiki plants under salt treatment did not suffer severe growth damages, but several leaves turned brown and fell off. Royal was the unique cultivar with low damage and few brown leaves, with plants somewhat similar to the control ones. These observations have confirmed the tolerance of this cultivar to stress conditions [10,11].

The Na^+^ and K^+^ ions concentration in the leaves of examined plants were concordant with the level of susceptibility or tolerance to salt stress of cultivars. Despite the high level of Na^+^ in the plant, Royal cultivar was able to tolerate salt stress, indicating that its elevated salinity tolerance was not a consequence of Na^+^ exclusion. In a previous experiment conducted by the authors [10], it was shown that, with respect to Koroneiki, Royal cultivar accumulated a high concentration of salt in the roots, showing that Royal salt tolerance may derive from its ability to protect the above ground meristematic tissues from the accumulations of Na^+^. In glycophytes, excessive Na^+^ often leads to K^+^ deficiency under salt stress [16]. In fact, K^+^ concentration decreased in all three cultivars except Royal, that maintained a high K^+^/Na^+^ concentration in the leaves, an important mechanism used by plants to adapt to salt stress [16,17,18,21]. The susceptible cultivars Fadak86 and Picual, with a high concentration of Na+ in their leaves, did not activate the mechanisms to prevent salt translocation or decreasing its transport and did not exclude Na^+^ from leaves, replacing it with K^+^, while the cultivar Koroneiki, partially salt susceptible, with the lowest concentration of Na^+^ in the leaves, seemed to apply the mechanism of compartmentalizing toxic ions within the leaves [22,61].

*NHX* genes induce enhanced Na^+^ accumulation in wild tomato when grown in the presence of NaCl [62]. The upregulation of *OeNHX7* in Royal and Fadak86 olive plants under stress at different time points was in accordance with Wu et al. [32], reporting that *NHX* genes were significantly upregulated by salt both in roots and leaves, and the transcription levels under high salinity were significantly higher than those under low or moderate salinity. The upregulation of *OeNHX7* gene at the end of the experiment (240 DATS) in Fadak86 plants could not activate any defense mechanisms against salt and plants harshly collapsed at that stage, while upregulation of this gene in salt treated Royal plants at 210 DATS, may have counteracted saline stress by sequestering Na^+^ in vacuoles, a process carried out by Na^+^/H^+^ exchangers that keep the cytosolic Na^+^ concentration low [24]. The *OeNHX6* gene completely failed to be responsive to salt stress. Effectively, also in treated plants of *Medicago truncatula*, *NHX6* did not show any significant expression with respect to the control [24]. Moreover, the not significant response of this gene to the high level of salt could depend on the tissue-specific function of *OeNHX6*.

*P5CS* gene expression is directly related to salt stress and ABA function in *O. sativa* [63,64,65]. *P5CS* gene expression level and the proline content increased in *Lepidium draba* with increasing concentrations of NaCl [63], and *P5CS1* and *P5CS2* increased salt tolerance in *Panicum virgatum* [64]. In a study of transcriptome analysis of olive [53], ATP binding activity has been observed in three transcripts differentially expressed under salt stress. Over-expression of *OeP5CS* gene at 210 DATS in both tolerant and moderately tolerant Royal and Koroneiki cultivars, highlighted this gene as one of the most solid candidates regulating salt tolerance. Our results confirmed what was previously observed in *Panicum virgatum* by Guan et al. [65]. Moreover, the regulatory elements detection in upstream/downstream parts of this gene did not show any differences among the four analyzed genomes, highlighting the strong conservation of UTRs regions. The increase in expression of *P5CS* by increasing salt concentration to 300 mM was also reported [66]. Moreover, several studies have demonstrated that over-expression of *P5CS* gene increases proline production and confers salt tolerance on transgenic plants [46]. For this reason, it is conceivable that *P5CS* and proline synthesis are involved in regulation of Na^+^ accumulation in leaves and, consequently, in salt stress tolerance [65].

The cysteine protease genes *RD21A* and *RD19A*, belonging to the papain family, were induced by water deficit and were responsive to salt stress in *Arabidopsis thaliana* [40]. Functional analysis of these proteases has shown that cysteine proteases play an important role in the programmed cell-death pathway during stress [67]. Effectively, in our study, *OeRD19A* was upregulated only in the salt tolerant cvs. Royal and Koroneiki at 210 DATS, strongly supporting the role of this gene to increase salt tolerance also in olive. Detection of only one polymorphism in 3′ UTR region of cvs. Farga and Leccino genomes confirmed the high conservation of *OeRD19A* in different olive cultivars. These results are in accordance with Qin et al. [41], reporting that the upregulation of this gene in tobacco transgenic plants enhanced their tolerance to salt stress, and, more importantly, this gene was up-regulated in *Arabidopsis pumila* under 500 mM NaCl [42]. This evidence makes this gene one of the strongest candidates to induce salt tolerance in olive.

*PetD* (CytB6) chloroplast gene is a component of the plastoquinone-plastocyanin reductase, involved in electron transport and generation of ATP. Under salinity stress, CytB6 and other chloroplast protein complexes were upregulated [50,68] as reported also in *Triticum aestivum* under salt stress [69]. In the study on the transcriptome response to salinity in olive [52], the same gene was upregulated in the stressed plants of cv. Kalamon. The upregulation of this gene could represent a defense measure to combat the negative oxidative damage caused by salt stress. The *OePetD* analyzed in this study showed a clear upregulation in the most tolerant cultivar Royal and, to a lesser extent, in Koroneiki, both at 210 DATS, confirming previous evidence [10]. The upregulation of this gene in the susceptible Picual cultivar during the recovery phase, could depend on the sprouting of new leaves and the increase of activity of this chloroplast gene.

*PI4Kg4* gene plays a regulatory role in increasing Ca^2+^ concentration in all parts of stressed plants, thus limiting the toxic effect of Na^+^ on the integrity of the plasma membrane [59] and contributing to Na^+^ exclusion [8]. The slight upregulation of *OePI4Kg4* in the most tolerant and in the most susceptible cultivar can be explained by the fact that the gene is activated in the presence of salt but, at least in olive, *OePI4Kg4* does not contribute to increase the ability of the plant to tolerate stress. This result confirmed what was observed in a previous work on olive [10], while the over-expression of this gene, even if significant among stressed and control plants, was not particularly high. The high number of REs (38) detected in the UTR regions can also justify the high polymorphism in this gene in different cultivars, which can affect gene expression. Moreover, the upregulation of this gene during recovery in cv. Koroneiki, could depend on the recovery of plant functions.

The interaction between CBL-CIPK (CBL-interacting protein kinase) regulates the activity of a series of transport proteins involved in the absorption and translocation of K^+^ and Na^+^, keeping the right balance of these cations in plants under stress conditions [33,70]. The role of *OeCBL3* to maintain the balance in K^+^ and Na^+^ ions in olive is controversial. In fact, the maximum expression was obtained in the most susceptible cultivar (Fadak86), which however showed the strongest reduction of K^+^ ions compared to the other cultivars and the highest stress damages. The upregulation of this gene at 210 DATS in a medium-tolerant cultivar such as Koroneiki, could support its effectiveness in controlling the K^+^/Na^+^ ratio of leaves, as in the tolerant cultivar Royal.

Among several zinc finger proteins belonging to the *BBX* proteins family, two are known to induce salt tolerance [49,50,71]. The *OeBBX19* analyzed in this study effectively showed over-expression in Royal plants at 210 DATS, confirming the effect of this gene to increase salt tolerance, but its upregulation in the most susceptible and damaged cultivar Fadak86 at 240 DATS could not activate salt tolerance mechanisms, likely when irreversible damages in the plant have already occurred. In the study of olive transcriptome under salt stress, two zinc ion binding transcripts were differentially expressed in salt-stressed plants [53].

*PIPs* improve Na^+^ exclusion in roots, tissue compartmentalization of Na^+^, water uptake by roots and leaf cell hydration [36]. A *PIP1* subgroup of the AQP genes, was upregulated in response to NaCl and able to increase root elongation under salt stress with elevated Na^+^ and K^+^ content [23]. The study on the AQP response to salt stress in tomato showed *PIP1;1*, *PIP1;3*, *PIP1;7*, *PIP2;10* and *PIP2;12* played an important role in regulating water transport under salt stress. Results suggest that *PIPs* were involved in mediating water transport in tomato plants, and the regulation of *PIP* expression under salt stress was associated with tissue type and stress duration [72]. But in our study, *OePIP1.1* did not show a clear pattern of expression associated with the ability to tolerate salt stress in olive, confirming its ambiguous role in salt stress response shown in a previous work, even if was demethylated under salt stress in susceptible cultivars [10]. The studies demonstrate that the expression response of AQPs is quite variable in different tissues, different plants and in different stress conditions (such as stress duration), and different AQP isoforms exhibited different responses even to the same stress condition [73,74,75].

Arabidopsis *SRP* homologs play a positive role in tissue growth and development and are related with cell wall organization, especially in the biogenesis of lipid droplets (LD), and *SRP1* and *SRP3* were induced by abiotic stresses like drought, low temperature and high salinity [45]. The involvement of SRPs and their upregulation under salinity stress was reported also by Hosseini et al. [76] and Fercha et al. [77]. Unfortunately, also *OeSRP* showed doubtful expression patterns in olive, with upregulation in both tolerant and susceptible cultivars. Probably, it could play a role in salt response that does not involve changes in tolerance.

## 4. Materials and Methods

### 4.1. Plant Material and Experimental Design

Four olive cultivars, with different tolerance to salt stress, have been studied in this experiment: Royal, as a high-tolerant cultivar [10,11], Koroneiki and Picual, selected as medium salt-tolerant cultivars [10,78] and Fadak86, as a susceptible cultivar [11]. Two-years old plants, average height 1.3–1.5 m, were grown in the greenhouse in 2.5 L plastic pots containing a substrate composed of 60% peat and 40% pumice (*w*/*w*). Twenty replicates/treatment/cultivar were considered, and, from these replicates, five plants were selected for destructive measurements Plants were exposed to natural light and an automatic ventilation system avoided to exceed a temperature of 35 °C. The minimum and maximum daily temperatures ranged between 9.4 and 15.2 °C, and 10.4–30.4 °C, respectively. Two treatments were applied: control (0) and 200 mM NaCl. Plants were irrigated three times a week, for three months, using salt-free half-strength Hoagland solution and for subsequent eight months from October, with half-strength Hoagland solution containing 200 mM NaCl.

In order to prevent osmotic shock, salt concentration was increased daily by 25 mM, to reach the 200 mM level, maintained along the entire experiment. Electrical Conductivity (EC) was determined weekly in the leaching solution with the conductometer Hanna Instruments- HI 9033, with values of about 1.2 and 21.4 dS m^−1^, in relation to 0 and 200 mM NaCl, respectively. EC measurement confirmed that an irrigation rate with a leaching fraction of 20–30% ensured a stable salinity level in pots throughout the course of the experiment. After treatment completion, stressed plants were irrigated with salt-free half-strength Hoagland solution for 21 days to recover plants from the stress.

### 4.2. Plant Growth and Chemical Measurements

Plant size was measured at the beginning and the end of experiment (240 DATS). Plant growth measurement of Koroneiki, Royal and Fadak86 cultivars are from the previous published study [11], those of cv. Picual are de novo provided in the present study.

In order to measure the content of sodium (Na^+^), and potassium (K^+^) ions, leaf tissue of control and 200 mM NaCl treated plants was collected at 240 DATS and dried at 65 °C for 48 h, finely ground and extracted with diluted nitric acid. The concentration of Na^+^ and K^+^ of leaf extract was measured through a flame photometer (Digiflame, GDV) [60,79].

### 4.3. Molecular Characterization of Salt Responsive Genes in Olive

The partial or full-length mRNA sequences, previously identified in olive [10,55], were used as queries to identify the genomic scaffolds in a BLAST search on four different olive genomes: cv. Leccino (http://olgenome.crea.gov.it/index.php/en/ (accessed on 23 December 2021)), cv. Farga (https://denovo.cnag.cat/olive (accessed on 23 December 2021)) [80], wild olive (*Olea europaea* var. *sylvestris*, http://olivegenome.org/ (accessed on 23 December 2021)) [81] and cv. Picual (https://genomaolivar.dipujaen.es/db/ (accessed on 23 December 2021)) [56]. Gene sequences and transcripts with best ID, E-values and hit scores were identified, followed by a nucleotide BLAST search in the NCBI database (https://blast.ncbi.nlm.nih.gov/Blast.cgi (accessed on 23 December 2021)) and the alignment with gene orthologs. BioEdit software (https://bioedit.software.informer.com/ (accessed on 23 December 2021)) was used to align gene sequences, distinguishing exons and introns. ORFs and corresponding proteins were predicted using the ExPASy translate tool (http://web.expasy.org/translate/ (accessed on 23 December 2021)) and fgenesh (http://www.softberry.com/berry.phtml (accessed on 23 December 2021)). Predicted olive proteins and their putative orthologues were aligned through ClustalW (https://bioedit.software.informer.com/ (accessed on 23 December 2021)). Protein homology with other plant species was verified by BLASTP in the non-redundant protein sequences database of NCBI. TAIR (https://www.arabidopsis.org/Blast (accessed on 23 December 2021)) and UNIPROT database resources (http://www.uniprot.org/blast (accessed on 23 December 2021)) were used to search gene ontology terms. Regulatory elements (RE) were predicted for each complete predicted or published gene where polymorphism among the four genomes in upstream/downstream part was found. The analysis was performed by the program Nsite, version 5 [82] with search parameters including: expected mean number 0.010, statistical significance level 0.950, level of homology between known RE and motif 80% and variation of distance between RE blocks 20%.

The complete sequence of each gene was amplified on DNA (25 ng) of cvs. Royal, Koroneiki, Picual and Fadak86 (using cv. Leccino as reference genome), by using LA Taq polymerase (Takara Bio Company), with the following PCR program: 98 °C for three min; 50 cycles of 98 °C for 25 s, 58 °C for 25 s, 68 °C for four min and final extension at 68 °C for 10 min. In order to verify sequence homology, portions of these genes were sequenced using BigDye Terminator v.3.1 Cycle Sequencing Kit (Applied Biosystems, Foster, CA, USA). Purified PCR products were sequenced on an ABI PRISM 3130 XL Genetic Analyzer (Applied Biosystems, Foster, CA, USA). The BioEdit 7.1.7 was used to align the sequenced fragments and identify polymorphisms within and between cultivars.

### 4.4. RNA Extraction, cDNA Synthesis and Gene Expression Analysis

Leaf samples were collected at 0, 180, 210 and 240 DATS, where 0 was the experiment start, exactly when salt concentration arrived at 200 mM in the treated plants (daily increase of 25 mM NaCl). After 210 DATS, two plants of each cultivar/treatment were placed in recovery condition, 14 and 21 days after recovery (14 and 21 DARS) their leaves were sampled for molecular analysis.

Total RNA was extracted from leaves by using the RNeasy Plant Mini Kit (Qiagen), according to manufacturer’s instructions. To avoid DNA contamination, each sample was treated with DNase I (Ambion, Thermo Fisher Scientific) and tested by amplifying the reference gene Elongation Factor 1 alpha (EF1α). Concentration of total RNA was assessed using a Nanodrop 2000c spectrophotometer (Thermo Fisher Scientific, DE, USA). Single-strand cDNA was synthesized from 500 ng of total RNA using oligo (dT)18 and SuperScript III Reverse Transcriptase (Thermo Fisher Scientific), according to manufacturer’s instructions. cDNA amplification was evaluated by PCR amplification of the EF1α gene.

Expression analyses were performed by RT-qPCR. Primers for the RT-qPCR experiments were designed using the program Primer3 version 4.0 (Table 3). Reactions were performed on three biological cDNA samples and three technical replicates for each sample by the same procedure reported in Mousavi et al. [10].

### 4.5. Statistical Analysis

Physiological and chemical data were analyzed using Two-way ANOVA followed by Tukey’s multiple comparisons test of GraphPad Prism version 9.0.0 (GraphPad Software, San Diego, CA, USA, www.graphpad.com (accessed on 23 December 2021)). Molecular data were analyzed by DAASTAT [83] using one-way ANOVA (**** *p* = 0.000, *** *p* < 0.001, ** *p* < 0.01 and * *p* < 0.05, *n* = 3), Tukey test was used to compare mean values.

## 5. Conclusions

In the present study, potted plants of four olive cultivars showing different levels of salt stress tolerance were subjected to strong salt stress (200 mM NaCl) for a long period under partially controlled conditions in the greenhouse. Plant growth measurements and observations on stress symptoms confirmed their tolerance level. The analysis of Na^+^ and K^+^ ions allowed an understanding of how the balance of these cations can influence the response to high salinity level in different cultivars. Sequence characterization and quantitative RT-qPCR analyses of the ten most interesting candidate genes, putatively involved in salt stress tolerance, based on evidence obtained in other species or directly from previous studies on olive, demonstrated that only four of them, namely *OeNHX7*, *OeP5CS*, *OeRD19A* and *OePetD*, showed a pattern of expression highly consistent with the level of salt tolerance of the olive cultivars. These genes, as well as most of the other six, were fully characterized and positioned on the olive linkage groups of cv. Leccino. The identification of salt stress responsive genes in olive represents a new resource for the genomics-assisted breeding and for genome editing. We strongly support olive phenomics and genomics as strategic tools for improving adaptation of olive to environmental stress and to overcome future scenarios of climate change.

## Figures and Tables

**Table 1:** Identified genes and corresponding transcripts, proteins and chromosome position in *Olea europaea* cv. Leccino genome.

Gene Name	Genotype	Genomic Scaffold	mRNA/CDS	Protein	Chromosome/Linkage Group	Comment	Accession Number
*OeNHX7*	Farga	Oe6_s07827	predicted	predicted			OK637283
	var. *sylvestris*	NC_036248.1	published	predicted	12		XM_023036083.1
	Leccino partial	tig00005075	predicted	-	6		
	Picual partial	Oleur061Scf1390, Oleur061Scf2766	predicted partial	-			[56]
*OeNHX6*	Farga	Oe6_s09856	predicted	predicted			OK637277
	var. *sylvestris*	NC_036249.1	published	published	13		XM_023036540.1
	Leccino partial	scaffold75631, scaffold60078, scaffold108620	predicted partial	predicted partial	8		
	Picual partial	Oleur061Scf1057	predicted	predicted		Published cds do not correspond to the other three genomes	[56]
*OeP5CS*	Farga	Oe6_s02546	predicted	predicted			OL310482
	var. *sylvestris*	CM008527.1	published	published	13		XM_023036864.1
	Leccino	scaffold44052 + 94205.1	predicted	predicted	8		OK637278
	Picual	Oleur061Scf2321	predicted	predicted		Published cds are partial	[56]
*OePIK4g*	Farga	Oe6_s03937	predicted	predicted			OL310483
	var. *sylvestris* partial	CM008529.1	not complete	not complete	15		XM_023040706.1
	Leccino	scaffold20411.1	published	published	23 *		MF958940.1
	Picual	Oleur061Scf0433	predicted	predicted		Published cds do not correspond to the other two genomes	[56]
*OeCBL3*	Farga	Oe6_s03919	predicted	predicted			OK637279
	var. *sylvestris* partial	CM008521.1	published	published	7	Published protein does not correspond to the other three genomes	XM_023001857.1
	Leccino partial	scaffold19373	predicted partial	predicted partial	8		
	Picual	Oleur061Scf1775	predicted	predicted		Published cds is partial	[56]
*OeBBX19*	Farga	Oe6_s01951	predicted	predicted			OL310484
	var. *sylvestris*	NC_036241.1	published	published	5		XM_023020441.1
	Leccino	scaffold28640.1	predicted	predicted	23 *		OK637280
	Picual partial	Oleur061Scf0258	predicted partial	predicted partial		Published cds and protein do not correspond to the three genomes	[56]
*OeRD19A*	Farga	Oe6_s05921	predicted	predicted			OL310485
	not find in var. *sylvestris*						XM_023001197.1
	Leccino	scaffold33560.1	predicted	predicted	6		OK637281
	not find in Picual		-	-			
*OePIP1.1*	Farga	Oe6_s00807	predicted	predicted			OL310486
	var. *sylvestris*	NC_036255.1	published	published	19		XM_022990009.1
	Leccino	scaffold116227.1	published	published	14 *		MF784562
	Picual	Oleur061Scf2846	predicted	predicted		Published cds and protein are partial	[56]
*OeSRP*	Farga	Oe6_s02151	predicted	predicted			OL310487
	var. *sylvestris*	NC_036256.1	published	published	20		XM_022991523.1
	Leccino	scaffold70781	predicted	predicted	17		OK637282
	Picual	Oleur061Scf3856	published	published			[56]
*OePetD*	Leccino	scaffold168451	published	published		Chloroplast gene	GU931818.1

* The scaffolds where the gene is located were mapped in different linkage groups, here was reported the most frequent linkage group based on the position of the gene.

**Table 2:** Identified genes, their structure and putative biological process.

Gene Name	Genomic Sequence Length (bp)	Transcript Sequence Length (bp)	CDS Sequence Length (bp)	Peptide Sequence Length	Molecular Function	Biological Process
*OeNHX7* *	58,522	2940	2646	882	Potassium/proton and sodium/proton antiporter activity	Potassium ion transmembrane transport; regulation of intracellular pH; regulation of reactive oxygen species metabolic process; response to hydrogen peroxide; response to oxidative stress; response to reactive oxygen species; response to salt stress; sodium ion imports across plasma membrane sodium ion transport
*OeNHX6* *	7410	2087	1572	524	Potassium/proton and sodium/proton antiporter activity	Potassium ion transmembrane transport; regulation of intracellular pH; sodium ion imports across plasma membrane
*OeP5CS*	6877	2570	1893	631	ATP binding; delta1-pyrroline-5-carboxylate synthetase activity; glutamate 5-kinase activity; glutamate-5-semialdehyde dehydrogenase activity	Hyperosmotic salinity response; L-proline biosynthetic process; pollen development; proline biosynthetic process; response to oxidative stress; response to salt stress; response to water deprivation; root development
*OePIK4g*	5122	3576	1743	581	1-phosphatidylinositol 4-kinase activity; ATP binding; protein serine/threonine kinase activity	Cellular response to hypoxia; phosphatidylinositol phosphorylation; protein autophosphorylation; response to salt stress
*OeCBL3* *	3887	1661	675	225	Calcium ion binding; kinase binding	Detection of calcium ion; potassium ion homeostasis
*OeBBX19*	3175	993	648	216	Zinc ion binding	Negative regulation of photo-morphogenesis; photo-morphogenesis; regulation of transcription, DNA-templated
*OeRD19A*	2471	1368	1131	377	Cysteine-type endopeptidase activity	Defense response to bacterium; proteolysis involved in cellular protein catabolic process; response to osmotic stress; response to salt stress
*OePIP1.1*	2109	1465	858	286	Water channel activity	Response to water deprivation; water transport
*OeSRP*	1347	1059	750	250	No information in uniprot	Developmental vegetative growth; lipid droplet organization; pollen development; positive regulation of growth; positive regulation of response to water deprivation
*OePetD*	160	-	159	53	Electron transporter, transferring electrons within cytochrome b6/f complex of photosystem II activity; electron transporter, transferring electrons within the cyclic electron transport pathway of photosynthesis activity	Photosynthetic electron transport chain

* These three genes were predicted from the published scaffolds of cv. Farga, while for other seven genes the reference genome was cv. Leccino.

**Table 3:** Specific primers used for quantitative expression of ten candidate transcripts.

Gene Name	Primer 5′-3′	Amplified Lenght (bp)	Position on the Gene Sequence
*OeNHX7*	Fw-GGCGCATATTGGAATACACGA	112	Exon 20
	Rev-GCTGACTGGCCTACTGTTAAGA		
*OeNHX6*	Fw-CAGAAGGGCTTGGTCTCTCC	382	Exon 13–15
	Rev-CATAGCTGGTCCCATGTCGG		
*OeP5CS*	Fw-GGGAAAGGAGGCCAGAAGAT	202	Exon 8–9
	Rev-GGGACTCATTGGACTGGTGA		
*OePIK4g*	Fw-AGTTCTGGTTAGGTGCCTGC	167	Exon 1
	Rev-TGCGGTCTTGGATATGAGGA		
*OeCBL3*	Fw-TGAAACCTTGTTGCTTGAGATCA	200	Exon 4–5
	Rev-GGATGGAATACAGAAAGTGCACG		
*OeBBX19*	Fw-CTCAATGCCAGACCTCAACG	260	Exon 4–5
	Rev-TGGCAATCATCATGAAGGTGC		
*OeRD19A*	Fw-TCCACAAGCTGCTGTTCACT	82	Exon 4
	Rev-CAGCGCTCCGGTTGTACTAA		
*OePIP1.1*	Fw-AAATCCGGCAGTGACTTTCG	92	Exon 2
	Rev-GATGCAGTGTCTTGGAGCCA		
*OeSRP*	Fw-CCATTGGTAGAAACAGCCGG	93	Exon 1
	Rev-GCAGGTAATACGACAGCGGA		
*OePetD*	Fw-AATGATCCTGTATTAAGAGCT	307	Chloroplast gene
	Rev-CTGCGGGATTATTAACAGTA		

## Data Availability

The data presented in this study are available in here.

## Supplementary Materials

The following are available online at www.mdpi.com/article/10.3390/ijms23010154/s1.
