# Supplementary material for: Characterization of Differentially Expressed Genes under Salt Stress in Olive
Source: Int J Mol Sci. 2021 Dec 23;23(1):154. doi: 10.3390/ijms23010154 (PMC8745295; doi:10.3390/ijms23010154)

Supplementary Material

Characterization of differentially expressed genes under salt stress in olive

Soraya Mousavi [1*], Roberto Mariotti [1], Maria Cristina Valeri [1], Luca Regni [2], Emanuele Lilli [1], Emidio Albertini [2], Primo Proietti [2], Daniela Businelli [2], Luciana Baldoni [1]

[1] Institute of Biosciences and Bioresources, National Research Council, 06128 Perugia, Italy; roberto.mariotti@ibbr.cnr.it (R.M.); mariacristinavaleri.mcv@gmail.com (M.C.V.); emanuele.lilli96@gmail.com (E.L.); luciana.baldoni@ibbr.cnr.it (L.B.)

[2] Department of Agricultural, Food and Environmental Sciences, University of Perugia, 06121 Perugia, Italy; luca.regni@unipg.it (L.R.); emidio.albertini@unipg.it (E.A.); primo.proietti@unipg.it (P.P.); daniela.businelli@unipg.it (D.B.)

[*] Correspondence: soraya.mousavi@ibbr.cnr.it; Tel.: +39-075-5014813

Table S1: The regulatory elements predicted by Nsite program in polymorphic UTRs of upstream/downstream parts of each gene in each cultivar.

***OeNHX6* cv. Farga 5′**

Motifs on "-" Strand: Mean Exp. Number 0.00737 Up.Conf.Int. 1 Found 1
27 CATGCAC 21 (Mism.= 0)

Motifs on "-" Strand: Mean Exp. Number 0.00591 Up.Conf.Int. 1 Found 1
174 AACAATCT 167 (Mism.= 0)

Motifs on "+" Strand: Mean Exp. Number 0.00786 Up.Conf.Int. 1 Found 1
155 CAAACaTACA 164 (Mism.= 1)

Motifs on "-" Strand: Mean Exp. Number 0.00069 Up.Conf.Int. 1 Found 1
101 GTTTTGGAA 93 (Mism.= 0)

Motifs on "+" Strand: Mean Exp. Number 0.00230 Up.Conf.Int. 1 Found 1
177 TtGAAtTTTCAG 188 (Mism.= 2)

A total of 5 different REs were found

***OeNHX6* var. *sylvestris* 5′**

Motifs on "-" Strand: Mean Exp. Number 0.00539 Up.Conf.Int. 1 Found 1
173 AACAATCT 166 (Mism.= 0)

Motifs on "-" Strand: Mean Exp. Number 0.00997 Up.Conf.Int. 1 Found 1
127 TCAACAAACaaA 116 (Mism.= 2)

Motifs on "-" Strand: Mean Exp. Number 0.00882 Up.Conf.Int. 1 Found 2
177 AACAAAC 171 (Mism.= 0)
125 AACAAAC 119 (Mism.= 0)

Motifs on "+" Strand: Mean Exp. Number 0.00374 Up.Conf.Int. 1 Found 1
189 CCCGGGGg 196 (Mism.= 1)

Motifs on "-" Strand: Mean Exp. Number 0.00366 Up.Conf.Int. 1 Found 1
31 TTCTaCAtGcCTTTTC 16 (Mism.= 3)

Motifs on "+" Strand: Mean Exp. Number 0.00222 Up.Conf.Int. 1 Found 1
176 TtGAAtTTTCAG 187 (Mism.= 2)

A total of 6 different REs were found

***OeNHX6* cv. Farga 3′**

Motifs on "-" Strand: Mean Exp. Number 0.00881 Up.Conf.Int. 1 Found 1
235 CCACTTG 229 (Mism.= 0)

Motifs on "+" Strand: Mean Exp. Number 0.00683 Up.Conf.Int. 1 Found 1
130 AGAAACAA 137 (Mism.= 0)

Motifs on "-" Strand: Mean Exp. Number 0.00213 Up.Conf.Int. 1 Found 1
202 ACTTcACACAC 192 (Mism.= 1)

Motifs on "-" Strand: Mean Exp. Number 0.00881 Up.Conf.Int. 1 Found 1
235 CCACTTG 229 (Mism.= 0)

Motifs on "+" Strand: Mean Exp. Number 0.00901 Up.Conf.Int. 1 Found 1
141 GTCAAAAT 148 (Mism.= 0)

A total of 5 different REs were found

***OeNHX6* var. *sylvestris* 3′**

Motifs on "-" Strand: Mean Exp. Number 0.00671 Up.Conf.Int. 1 Found 1
261 TGACGTAA 254 (Mism.= 0)

Motifs on "+" Strand: Mean Exp. Number 0.00385 Up.Conf.Int. 1 Found 1
227 ATcGaGTgGCTGTAC 241 (Mism.= 3)

Motifs on "-" Strand: Mean Exp. Number 0.00247 Up.Conf.Int. 1 Found 1
202 ACTTcACACAC 192 (Mism.= 1)

Motifs on "+" Strand: Mean Exp. Number 0.00760 Up.Conf.Int. 1 Found 1
141 GTCAAAAT 148 (Mism.= 0)

A total of 4 different REs were found

***OePI4KG* cv. Farga 5′**

Motifs on "-" Strand: Mean Exp. Number 0.00704 Up.Conf.Int. 1 Found 1
209 TCtACGTCTC 200 (Mism.= 1)

Motifs on "+" Strand: Mean Exp. Number 0.00771 Up.Conf.Int. 1 Found 1
200 GAgACGTAGA 209 (Mism.= 1)

Motifs on "+" Strand: Mean Exp. Number 0.00932 Up.Conf.Int. 1 Found 1
380 AgaATTGATTGAG 392 (Mism.= 2)

Motifs on "+" Strand: Mean Exp. Number 0.00999 Up.Conf.Int. 1 Found 1
15 TCAACGTGTC 24 (Mism.= 0)

Motifs on "+" Strand: Mean Exp. Number 0.00186 Up.Conf.Int. 1 Found 1
68 CTCCAACcAAaCC 80 (Mism.= 2)

Motifs on "-" Strand: Mean Exp. Number 0.00756 Up.Conf.Int. 1 Found 1

566 AAACCCTAA   558 (Mism.= 0)

Motifs on "+" Strand: Mean Exp. Number   0.00756   Up.Conf.Int. 1   Found   1
558 TTAGGGTTT   566 (Mism.= 0)

Motifs on "+" Strand: Mean Exp. Number   0.00084   Up.Conf.Int. 1   Found   1
71 CAACCAAACCAagT   84 (Mism.= 2)

Motifs on "-" Strand: Mean Exp. Number   0.00578   Up.Conf.Int. 1   Found   5
110 AGAGAGAGA   102 (Mism.= 0)
108 AGAGAGAGA   100 (Mism.= 0)
106 AGAGAGAGA   98 (Mism.= 0)
104 AGAGAGAGA   96 (Mism.= 0)
102 AGAGAGAGA   94 (Mism.= 0)

Motifs on "+" Strand: Mean Exp. Number   0.00817   Up.Conf.Int. 1   Found   1
65 ctTCTCCAACCA   76 (Mism.= 2)

Motifs on "+" Strand: Mean Exp. Number   0.00533   Up.Conf.Int. 1   Found   1
109 CtAACGTACA   118 (Mism.= 1)

Motifs on "+" Strand: Mean Exp. Number   0.00160   Up.Conf.Int. 1   Found   1
414 GAGGGTATTTTgGTcA   429 (Mism.= 2)

Motifs on "-" Strand: Mean Exp. Number   0.00246   Up.Conf.Int. 1   Found   4
113 GttAGAGAGAGAGAGAg   97 (Mism.= 3)
111 tAGAGAGAGAGAGAGAg   95 (Mism.= 2)
109 GAGAGAGAGAGAGAGAA   93 (Mism.= 0)
107 GAGAGAGAGAGAGAaAA   91 (Mism.= 1)

Motifs on "-" Strand: Mean Exp. Number   0.00863   Up.Conf.Int. 1   Found   1
393 TCTCAATC   386 (Mism.= 0)

Motifs on "-" Strand: Mean Exp. Number   0.00556   Up.Conf.Int. 1   Found   1
586 TTTTCCAC   579 (Mism.= 0)

Motifs on "-" Strand: Mean Exp. Number   0.00622   Up.Conf.Int. 1   Found   1
501 GCCATGTGAt   492 (Mism.= 1)

Motifs on "-" Strand: Mean Exp. Number   0.00773   Up.Conf.Int. 1   Found   1
517 tTACTCTACaC   507 (Mism.= 2)

Motifs on "-" Strand: Mean Exp. Number   0.00951   Up.Conf.Int. 1   Found   1
213 TtACTCTACGt   203 (Mism.= 2)

Motifs on "-" Strand: Mean Exp. Number   0.00209   Up.Conf.Int. 1   Found   1
129 TTTCTTGTTCT   119 (Mism.= 1)

Motifs on "-" Strand: Mean Exp. Number   0.00247   Up.Conf.Int. 1   Found   1
201 TCGTGTGC   194 (Mism.= 0)

Motifs on "+" Strand: Mean Exp. Number 0.00601 Up.Conf.Int. 1 Found 7
87 CTCatTtTCTCTCTCT 102 (Mism.= 3)
89 CatTtTCTCTCTCTCT 104 (Mism.= 3)
91 tTtTCTCTCTCTCTCT 106 (Mism.= 2)
93 tTCTCTCTCTCTCTCT 108 (Mism.= 1)
95 CTCTCTCTCTCTCTCT 110 (Mism.= 0)
97 CTCTCTCTCTCTCTaa 112 (Mism.= 2)
99 CTCTCTCTCTCTaaCg 114 (Mism.= 3)

Motifs on "+" Strand: Mean Exp. Number 0.00366 Up.Conf.Int. 1 Found 1
14 TTCAACGTGtCt 25 (Mism.= 2)

A total of 22 different REs were found

***OePI4KG* cv. Leccino 5′**

Motifs on "-" Strand: Mean Exp. Number 0.00684 Up.Conf.Int. 1 Found 1
211 TCtACGTCTC 202 (Mism.= 1)

Motifs on "+" Strand: Mean Exp. Number 0.00750 Up.Conf.Int. 1 Found 1
202 GAgACGTAGA 211 (Mism.= 1)

Motifs on "+" Strand: Mean Exp. Number 0.00906 Up.Conf.Int. 1 Found 1
382 AgaATTGATTGAG 394 (Mism.= 2)

Motifs on "+" Strand: Mean Exp. Number 0.00051 Up.Conf.Int. 1 Found 1
15 TCAACGTGTC 24 (Mism.= 0)

Motifs on "+" Strand: Mean Exp. Number 0.00185 Up.Conf.Int. 1 Found 1
68 CTCCAACcAAaCC 80 (Mism.= 2)

Motifs on "-" Strand: Mean Exp. Number 0.00765 Up.Conf.Int. 1 Found 1
568 AAACCCTAA 560 (Mism.= 0)

Motifs on "+" Strand: Mean Exp. Number 0.00765 Up.Conf.Int. 1 Found 1
560 TTAGGGTTT 568 (Mism.= 0)

Motifs on "+" Strand: Mean Exp. Number 0.00083 Up.Conf.Int. 1 Found 1
71 CAACCAAACCAagT 84 (Mism.= 2)

Motifs on "-" Strand: Mean Exp. Number 0.00609 Up.Conf.Int. 1 Found 6
112 AGAGAGAGA 104 (Mism.= 0)
110 AGAGAGAGA 102 (Mism.= 0)
108 AGAGAGAGA 100 (Mism.= 0)
106 AGAGAGAGA 98 (Mism.= 0)
104 AGAGAGAGA 96 (Mism.= 0)
102 AGAGAGAGA 94 (Mism.= 0)

Motifs on "+" Strand: Mean Exp. Number 0.00813 Up.Conf.Int. 1 Found 1
65 ctTCTCCAACCA 76 (Mism.= 2)

Motifs on "+" Strand: Mean Exp. Number 0.00519 Up.Conf.Int. 1 Found 1
111 CtAACGTACA 120 (Mism.= 1)

Motifs on "+" Strand: Mean Exp. Number 0.00158 Up.Conf.Int. 1 Found 1
416 GAGGGTATTTTgGTcA 431 (Mism.= 2)

Motifs on "-" Strand: Mean Exp. Number 0.00265 Up.Conf.Int. 1 Found 5
115 GttAGAGAGAGAGAGAg 99 (Mism.= 3)
113 tAGAGAGAGAGAGAGAg 97 (Mism.= 2)
111 GAGAGAGAGAGAGAGAg 95 (Mism.= 1)
109 GAGAGAGAGAGAGAGAA 93 (Mism.= 0)
107 GAGAGAGAGAGAGAaAA 91 (Mism.= 1)

Motifs on "-" Strand: Mean Exp. Number 0.00841 Up.Conf.Int. 1 Found 1
395 TCTCAATC 388 (Mism.= 0)

Motifs on "-" Strand: Mean Exp. Number 0.00532 Up.Conf.Int. 1 Found 1
588 TTTTCCAC 581 (Mism.= 0)

Motifs on "-" Strand: Mean Exp. Number 0.00622 Up.Conf.Int. 1 Found 1
503 GCCATGTGAt 494 (Mism.= 1)

Motifs on "-" Strand: Mean Exp. Number 0.00752 Up.Conf.Int. 1 Found 1
519 tTACTCTACaC 509 (Mism.= 2)

Motifs on "-" Strand: Mean Exp. Number 0.00936 Up.Conf.Int. 1 Found 1
215 TtACTCTACGt 205 (Mism.= 2)

Motifs on "-" Strand: Mean Exp. Number 0.00196 Up.Conf.Int. 1 Found 1
131 TTTCTTGTTCT 121 (Mism.= 1)

Motifs on "-" Strand: Mean Exp. Number 0.00242 Up.Conf.Int. 1 Found 1
203 TCGTGTGC 196 (Mism.= 0)

Motifs on "+" Strand: Mean Exp. Number 0.00644 Up.Conf.Int. 1 Found 8
87 CTCatTtTCTCTCTCT 102 (Mism.= 3)
89 CatTtTCTCTCTCTCT 104 (Mism.= 3)
91 tTtTCTCTCTCTCTCT 106 (Mism.= 2)
93 tTCTCTCTCTCTCTCT 108 (Mism.= 1)
95 CTCTCTCTCTCTCTCT 110 (Mism.= 0)
97 CTCTCTCTCTCTCTCT 112 (Mism.= 0)
99 CTCTCTCTCTCTCTaa 114 (Mism.= 2)
101 CTCTCTCTCTCTaaCg 116 (Mism.= 3)

Motifs on "+" Strand: Mean Exp. Number 0.00364 Up.Conf.Int. 1 Found 1
14 TTCAACGTGtCt 25 (Mism.= 2)

A total of 22 different REs were found

***OePIK4g* cv. Picual 5′**

Motifs on "-" Strand: Mean Exp. Number 0.00705 Up.Conf.Int. 1 Found 1
211 TCtACGTCTC 202 (Mism.= 1)

Motifs on "+" Strand: Mean Exp. Number 0.00762 Up.Conf.Int. 1 Found 1
202 GAgACGTAGA 211 (Mism.= 1)

Motifs on "+" Strand: Mean Exp. Number 0.00915 Up.Conf.Int. 1 Found 1
382 AgaATTGATTGAG 394 (Mism.= 2)

Motifs on "+" Strand: Mean Exp. Number 0.00051 Up.Conf.Int. 1 Found 1
15 TCAACGTGTC 24 (Mism.= 0)

Motifs on "+" Strand: Mean Exp. Number 0.00184 Up.Conf.Int. 1 Found 1
68 CTCCAACcAAaCC 80 (Mism.= 2)

Motifs on "-" Strand: Mean Exp. Number 0.00766 Up.Conf.Int. 1 Found 1
568 AAACCCTAA 560 (Mism.= 0)

Motifs on "+" Strand: Mean Exp. Number 0.00766 Up.Conf.Int. 1 Found 1
560 TTAGGGTTT 568 (Mism.= 0)

Motifs on "+" Strand: Mean Exp. Number 0.00082 Up.Conf.Int. 1 Found 1
71 CAACCAAACCAagT 84 (Mism.= 2)

Motifs on "-" Strand: Mean Exp. Number 0.00595 Up.Conf.Int. 1 Found 6
112 AGAGAGAGA 104 (Mism.= 0)
110 AGAGAGAGA 102 (Mism.= 0)
108 AGAGAGAGA 100 (Mism.= 0)
106 AGAGAGAGA 98 (Mism.= 0)
104 AGAGAGAGA 96 (Mism.= 0)
102 AGAGAGAGA 94 (Mism.= 0)

Motifs on "+" Strand: Mean Exp. Number 0.00824 Up.Conf.Int. 1 Found 1
111 CtAACGTACA 120 (Mism.= 1)

Motifs on "+" Strand: Mean Exp. Number 0.00807 Up.Conf.Int. 1 Found 1
65 ctTCTCCAACCA 76 (Mism.= 2)

Motifs on "+" Strand: Mean Exp. Number 0.00521 Up.Conf.Int. 1 Found 1
111 CtAACGTACA 120 (Mism.= 1)

Motifs on "+" Strand: Mean Exp. Number 0.00160 Up.Conf.Int. 1 Found 1
416 GAGGGTATTTTgGTcA 431 (Mism.= 2)

Motifs on "-" Strand: Mean Exp. Number 0.00257 Up.Conf.Int. 1 Found 5
115 GttAGAGAGAGAGAGAg 99 (Mism.= 3)
113 tAGAGAGAGAGAGAGAg 97 (Mism.= 2)
111 GAGAGAGAGAGAGAGAg 95 (Mism.= 1)
109 GAGAGAGAGAGAGAGAA 93 (Mism.= 0)

107 GAGAGAGAGAGAGAaAA   91 (Mism.= 1)

Motifs on "-" Strand: Mean Exp. Number   0.00853   Up.Conf.Int. 1   Found   1
395 TCTCAATC   388 (Mism.= 0)

Motifs on "-" Strand: Mean Exp. Number   0.00543   Up.Conf.Int. 1   Found   1
588 TTTTCCAC   581 (Mism.= 0)

Motifs on "-" Strand: Mean Exp. Number   0.00629   Up.Conf.Int. 1   Found   1
503 GCCATGTGAt   494 (Mism.= 1)

Motifs on "-" Strand: Mean Exp. Number   0.00769   Up.Conf.Int. 1   Found   1
519 tTACTCTACaC   509 (Mism.= 2)

Motifs on "-" Strand: Mean Exp. Number   0.00951   Up.Conf.Int. 1   Found   1
215 TtACTCTACGt   205 (Mism.= 2)

Motifs on "-" Strand: Mean Exp. Number   0.00198   Up.Conf.Int. 1   Found   1
131 TTTCTTGTTCT   121 (Mism.= 1)

Motifs on "-" Strand: Mean Exp. Number   0.00246   Up.Conf.Int. 1   Found   1
203 TCGTGTGC   196 (Mism.= 0)

Motifs on "+" Strand: Mean Exp. Number   0.00626   Up.Conf.Int. 1   Found   8
87 CTCatTtTCTCTCTCT   102 (Mism.= 3)
89 CatTtTCTCTCTCTCT   104 (Mism.= 3)
91 tTtTCTCTCTCTCTCT   106 (Mism.= 2)
93 tTCTCTCTCTCTCTCT   108 (Mism.= 1)
95 CTCTCTCTCTCTCTCT   110 (Mism.= 0)
97 CTCTCTCTCTCTCTCT   112 (Mism.= 0)
99 CTCTCTCTCTCTCTaa   114 (Mism.= 2)
101 CTCTCTCTCTCTaaCg   116 (Mism.= 3)

Motifs on "+" Strand: Mean Exp. Number   0.00369   Up.Conf.Int. 1   Found   1
14 TTCAACGTGtCt   25 (Mism.= 2)

A total of 23 different REs were found

***OePI4KG* cv. Farga 3′**

Motifs on "+" Strand: Mean Exp. Number   0.00381   Up.Conf.Int. 1   Found   1
295 TAGTGCTGT   303 (Mism.= 0)

Motifs on "-" Strand: Mean Exp. Number   0.00617   Up.Conf.Int. 1   Found   1
821 TCCGTGTAaCA   811 (Mism.= 1)

Motifs on "-" Strand: Mean Exp. Number   0.00229   Up.Conf.Int. 1   Found   1
1102 CCATTGTTTCGG   1091 (Mism.= 1)

Motifs on "+" Strand: Mean Exp. Number   0.00051   Up.Conf.Int. 1   Found   1

765 TgCACGTGcCACC   777 (Mism.= 2)

Motifs on "+" Strand: Mean Exp. Number   0.00584   Up.Conf.Int. 1   Found   1
766 GCACGTGC   773 (Mism.= 0)

Motifs on "-" Strand: Mean Exp. Number   0.00584   Up.Conf.Int. 1   Found   1
773 GCACGTGC   766 (Mism.= 0)

Motifs on "-" Strand: Mean Exp. Number   0.00189   Up.Conf.Int. 1   Found   1
640 TtGAAGTTTCAG   629 (Mism.= 1)

A total of 7 different REs were found

OePIK4g **cv. Leccino 3′**

Motifs on "+" Strand: Mean Exp. Number   0.00366   Up.Conf.Int. 1   Found   1
295 TAGTGCTGT   303 (Mism.= 0)

Motifs on "-" Strand: Mean Exp. Number   0.00600   Up.Conf.Int. 1   Found   1
805 TCCGTGTAaCA   795 (Mism.= 1)

Motifs on "-" Strand: Mean Exp. Number   0.00229   Up.Conf.Int. 1   Found   1
1086 CCATTGTTTCGG   1075 (Mism.= 1)

Motifs on "-" Strand: Mean Exp. Number   0.00191   Up.Conf.Int. 1   Found   1
640 TtGAAGTTTCAG   629 (Mism.= 1)

A total of 4 different REs were found

OePIK4g **cv. Picual 3′**

Motifs on "+" Strand: Mean Exp. Number   0.00372   Up.Conf.Int. 1   Found   1
295 TAGTGCTGT   303 (Mism.= 0)

Motifs on "-" Strand: Mean Exp. Number   0.00614   Up.Conf.Int. 1   Found   1
821 TCCGTGTAaCA   811 (Mism.= 1)

Motifs on "-" Strand: Mean Exp. Number   0.00234   Up.Conf.Int. 1   Found   1
1102 CCATTGTTTCGG   1091 (Mism.= 1)

Motifs on "+" Strand: Mean Exp. Number   0.00052   Up.Conf.Int. 1   Found   1
765 TgCACGTGcCACC   777 (Mism.= 2)

Motifs on "+" Strand: Mean Exp. Number   0.00583   Up.Conf.Int. 1   Found   1
766 GCACGTGC   773 (Mism.= 0)

Motifs on "-" Strand: Mean Exp. Number   0.00583   Up.Conf.Int. 1   Found   1
773 GCACGTGC   766 (Mism.= 0)

Motifs on "-" Strand: Mean Exp. Number 0.00191 Up.Conf.Int. 1 Found 1
640 TtGAAGTTTCAG 629 (Mism.= 1)

A total of 7 different REs were found

***OeBBX19* cv. Farga 5′**

Motifs on "+" Strand: Mean Exp. Number 0.00526 Up.Conf.Int. 1 Found 1
83 CTTTTGatTACCTTA 97 (Mism.= 2)

Motifs on "+" Strand: Mean Exp. Number 0.00295 Up.Conf.Int. 1 Found 1
35 gCATAAT 41 --23-- 65 ATTATAT 71 (Mism.= 1/ 0)

Motifs on "-" Strand: Mean Exp. Number 0.00680 Up.Conf.Int. 1 Found 1
115 GGTAATTA 108 (Mism.= 0)

Motifs on "-" Strand: Mean Exp. Number 0.00787 Up.Conf.Int. 1 Found 1
60 AgAACCATCCAc 49 (Mism.= 2)

Motifs on "+" Strand: Mean Exp. Number 0.00242 Up.Conf.Int. 1 Found 1
146 TAATTGGAaAAA 157 (Mism.= 1)

Motifs on "-" Strand: Mean Exp. Number 0.00602 Up.Conf.Int. 1 Found 1
165 ATTACGTaTT 156 (Mism.= 1)

A total of 6 different REs were found

***OeBBX19* cv. Leccino 5′**

Motifs on "+" Strand: Mean Exp. Number 0.00557 Up.Conf.Int. 1 Found 1
83 CTTTTGatTACCTTA 97 (Mism.= 2)

Motifs on "+" Strand: Mean Exp. Number 0.00306 Up.Conf.Int. 1 Found 1
35 ACtTAAT 41 --23-- 65 ATTATAT 71 (Mism.= 1/ 0)

Motifs on "-" Strand: Mean Exp. Number 0.00738 Up.Conf.Int. 1 Found 1
113 GGTAATTA 106 (Mism.= 0)

Motifs on "-" Strand: Mean Exp. Number 0.00474 Up.Conf.Int. 1 Found 1
164 AATGACGTatTTT 152 (Mism.= 2)

Motifs on "+" Strand: Mean Exp. Number 0.00643 Up.Conf.Int. 1 Found 1
26 CAAACGgACA 35 (Mism.= 1)

Motifs on "-" Strand: Mean Exp. Number 0.00692 Up.Conf.Int. 1 Found 1
60 AgAACCATCCAc 49 (Mism.= 2)

Motifs on "+" Strand: Mean Exp. Number 0.00223 Up.Conf.Int. 1 Found 1
144 TAATTGGAaAAA 155 (Mism.= 1)

Motifs on "-" Strand: Mean Exp. Number 0.00041 Up.Conf.Int. 1 Found 1
166 TTaATGACGTAtTT 153 (Mism.= 2)

Motifs on "-" Strand: Mean Exp. Number 0.00236 Up.Conf.Int. 1 Found 1
164 aATGACGTATTt 153 (Mism.= 2)

Motifs on "+" Strand: Mean Exp. Number 0.00780 Up.Conf.Int. 1 Found 1
155 aTACGTCATTaA 166 (Mism.= 2)

A total of 10 different REs were found

***OeBBX19* cv. Farga 3′**

Motifs on "-" Strand: Mean Exp. Number 0.00870 Up.Conf.Int. 1 Found 1
142 aACAGTGTTAa 132 (Mism.= 2)

Motifs on "+" Strand: Mean Exp. Number 0.00307 Up.Conf.Int. 1 Found 1
99 cAcCTGACCTTT 110 (Mism.= 2)

Motifs on "-" Strand: Mean Exp. Number 0.00342 Up.Conf.Int. 1 Found 1
94 CGATTTAAGG 85 (Mism.= 1)

A total of 3 different REs were found

***OeBBX19* cv. Leccino 3′**

Motifs on "-" Strand: Mean Exp. Number 0.00896 Up.Conf.Int. 1 Found 1
142 aACAGTGTTAa 132 (Mism.= 2)

Motifs on "+" Strand: Mean Exp. Number 0.00308 Up.Conf.Int. 1 Found 1
99 cAcCTGACCTTT 110 (Mism.= 2)

Motifs on "-" Strand: Mean Exp. Number 0.00949 Up.Conf.Int. 1 Found 1
97 CAATgATTT 89 (Mism.= 1)

A total of 3 different REs were found

***OeRD19A* cv. Farga 3′**

Motifs on "-" Strand: Mean Exp. Number 0.00527 Up.Conf.Int. 1 Found 1
28 GGaAATTA 21 (Mism.= 1)

Motifs on "+" Strand: Mean Exp. Number 0.00984 Up.Conf.Int. 1 Found 1
19 aATAATTT 26 (Mism.= 1)

A total of 2 different REs were found

***OeRD19A* cv. Leccino 3′**

Motifs on "-" Strand: Mean Exp. Number 0.00412 Up.Conf.Int. 1 Found 1
28 GGaAATTA 21 (Mism.= 1)

Motifs on "-" Strand: Mean Exp. Number 0.00801 Up.Conf.Int. 1 Found 1
26 aAATTATTT 18 (Mism.= 1)

Motifs on "+" Strand: Mean Exp. Number 0.00765 Up.Conf.Int. 1 Found 1
19 aATAATTT 26 (Mism.= 1)

A total of 3 different REs were found

***OePIP1.1* cv. Farga 5′**

Motifs on "+" Strand: Mean Exp. Number 0.00478 Up.Conf.Int. 1 Found 1
50 ATAtAAATCAA 60 (Mism.= 1)

Motifs on "-" Strand: Mean Exp. Number 0.00268 Up.Conf.Int. 1 Found 1
151 CTCACACT 144 (Mism.= 0)

Motifs on "+" Strand: Mean Exp. Number 0.00170 Up.Conf.Int. 1 Found 1
150 AGAAAGAGA 158 (Mism.= 0)

Motifs on "-" Strand: Mean Exp. Number 0.00418 Up.Conf.Int. 1 Found 1
72 AAGCGgAAGT 63 (Mism.= 1)

Motifs on "-" Strand: Mean Exp. Number 0.00534 Up.Conf.Int. 1 Found 1
145 CTAAAACAAG 136 (Mism.= 1)

A total of 5 different REs were found

***OePIP1.1* cv. Leccino 5′**

Motifs on "+" Strand: Mean Exp. Number 0.00530 Up.Conf.Int. 1 Found 1
50 ATAtAAATCAA 60 (Mism.= 1)

Motifs on "-" Strand: Mean Exp. Number 0.00291 Up.Conf.Int. 1 Found 1
151 CTCACACT 144 (Mism.= 0)

Motifs on "+" Strand: Mean Exp. Number 0.00190 Up.Conf.Int. 1 Found 1
150 AGAAAGAGA 158 (Mism.= 0)

Motifs on "-" Strand: Mean Exp. Number 0.00394 Up.Conf.Int. 1 Found 1
72 AAGCGaAAGT 63 (Mism.= 1)

Motifs on "-" Strand: Mean Exp. Number 0.00801 Up.Conf.Int. 1 Found 1
89 TGTGATG 83 (Mism.= 0)

Motifs on "-" Strand: Mean Exp. Number 0.00576 Up.Conf.Int. 1 Found 1
145 CTAAAACAAG 136 (Mism.= 1)

A total of 6 different REs were found

***OePIP1.1* cv. Picual 5′**

Motifs on "+" Strand: Mean Exp. Number 0.00435 Up.Conf.Int. 1 Found 1
50 ATAtAAATCAA 60 (Mism.= 1)

Motifs on "-" Strand: Mean Exp. Number 0.00258 Up.Conf.Int. 1 Found 1
151 CTCACACT 144 (Mism.= 0)

Motifs on "+" Strand: Mean Exp. Number 0.00151 Up.Conf.Int. 1 Found 1
150 AGAAAGAGA 158 (Mism.= 0)

Motifs on "-" Strand: Mean Exp. Number 0.00438 Up.Conf.Int. 1 Found 1
72 AAGCGgAAGT 63 (Mism.= 1)

Motifs on "-" Strand: Mean Exp. Number 0.00543 Up.Conf.Int. 1 Found 1
145 CTAAAACAAG 136 (Mism.= 1)

A total of 5 different REs were found

***OePIP1.1* cv. Farga 3′**

Motifs on "+" Strand: Mean Exp. Number 0.00408 Up.Conf.Int. 1 Found 1
202 AAAGTGGGGaCt 213 (Mism.= 2)

Motifs on "-" Strand: Mean Exp. Number 0.00483 Up.Conf.Int. 1 Found 1
131 TgTCACCAtCTA 120 (Mism.= 2)

Motifs on "-" Strand: Mean Exp. Number 0.00931 Up.Conf.Int. 1 Found 1
145 CTTTTCTAAG 136 (Mism.= 1)

Motifs on "+" Strand: Mean Exp. Number 0.00251 Up.Conf.Int. 1 Found 1
148 CtGAACTTTCAA 159 (Mism.= 1)

Motifs on "+" Strand: Mean Exp. Number 0.00564 Up.Conf.Int. 1 Found 1
44 AGCCCAT 50 (Mism.= 0)

A total of 5 different REs were found

***OePIP1.1* cv. Leccino 3′**

Motifs on "+" Strand: Mean Exp. Number 0.00409 Up.Conf.Int. 1 Found 1
202 AAAGTGGGGaCt 213 (Mism.= 2)

Motifs on "-" Strand: Mean Exp. Number 0.00508 Up.Conf.Int. 1 Found 1
131 TgTCACCAtCTA 120 (Mism.= 2)

Motifs on "-" Strand: Mean Exp. Number 0.00874 Up.Conf.Int. 1 Found 1
145 CTTTTCTAAG 136 (Mism.= 1)

Motifs on "+" Strand: Mean Exp. Number 0.00365 Up.Conf.Int. 1 Found 1
1 gCGATGACATTa 12 (Mism.= 2)

Motifs on "+" Strand: Mean Exp. Number 0.00246 Up.Conf.Int. 1 Found 1
148 CtGAACTTTCAA 159 (Mism.= 1)

Motifs on "-" Strand: Mean Exp. Number 0.00923 Up.Conf.Int. 1 Found 1
14 TTTAATGT 7 (Mism.= 0)

Motifs on "+" Strand: Mean Exp. Number 0.00546 Up.Conf.Int. 1 Found 1
44 AGCCCAT 50 (Mism.= 0)

A total of 7 different REs were found

***OeSRP* cv. Farga 5′**

Motifs on "-" Strand: Mean Exp. Number 0.00432 Up.Conf.Int. 1 Found 1
20 CATGCAA 14 (Mism.= 0)

Motifs on "-" Strand: Mean Exp. Number 0.00894 Up.Conf.Int. 1 Found 1
51 TCCGTGTttCA 41 (Mism.= 2)

Motifs on "-" Strand: Mean Exp. Number 0.00283 Up.Conf.Int. 1 Found 1
194 TTTTTAGTGGGTTTTT 179 (Mism.= 3)

Motifs on "+" Strand: Mean Exp. Number 0.00573 Up.Conf.Int. 1 Found 1
3 CCAAAATTTG 12 (Mism.= 1)

Motifs on "+" Strand: Mean Exp. Number 0.00191 Up.Conf.Int. 1 Found 1
3 CCAAAATTTG 12 (Mism.= 0)

Motifs on "-" Strand: Mean Exp. Number 0.00979 Up.Conf.Int. 1 Found 1
21 GCATGCAA 14 (Mism.= 1)

Motifs on "-" Strand: Mean Exp. Number 0.00192 Up.Conf.Int. 1 Found 1
177 TAGTACAC 170 (Mism.= 0)

A total of 7 different REs were found

***OeSRP* cv. Leccino 5′**

Motifs on "-" Strand: Mean Exp. Number 0.00451 Up.Conf.Int. 1 Found 1
20 CATGCAA 14 (Mism.= 0)

Motifs on "-" Strand: Mean Exp. Number 0.00984 Up.Conf.Int. 1 Found 1
51 TCCGTGTttCA 41 (Mism.= 2)

Motifs on "-" Strand: Mean Exp. Number 0.00267 Up.Conf.Int. 1 Found 1
190 TTTTTTGTGGGTTTTT 175 (Mism.= 3)

Motifs on "+" Strand: Mean Exp. Number 0.00590 Up.Conf.Int. 1 Found 1
3 CCAAAATTTG 12 (Mism.= 1)

Motifs on "+" Strand: Mean Exp. Number 0.00188 Up.Conf.Int. 1 Found 1
3 CCAAAATTTG 12 (Mism.= 0)

Motifs on "-" Strand: Mean Exp. Number 0.00198 Up.Conf.Int. 1 Found 1
173 TAGTACAC 166 (Mism.= 0)

A total of 6 different REs were found

Figure S1: The amino acid changes and their position in the five out of ten studied genes.

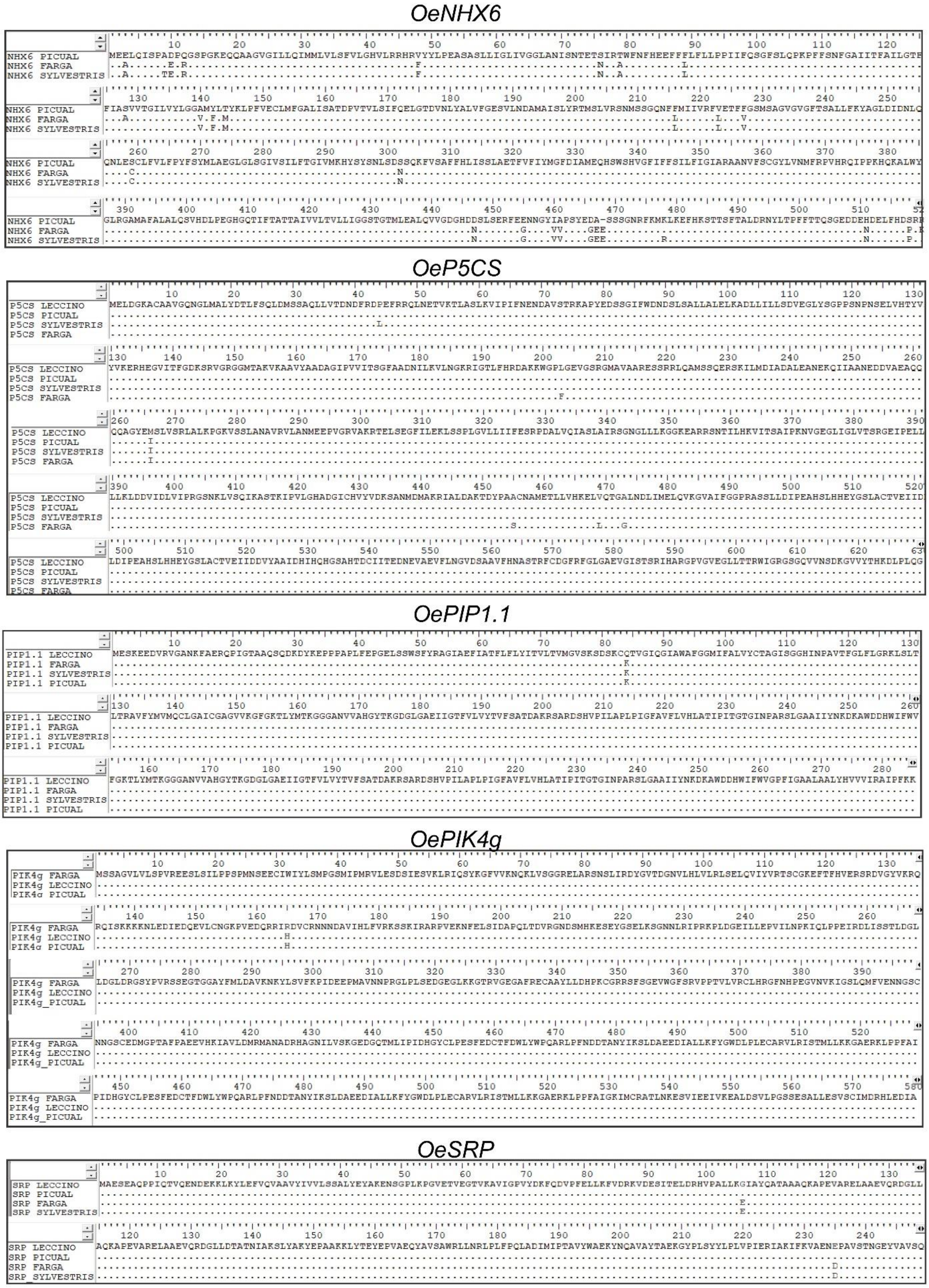

Supplement: Supplementary file 1 [file ijms-23-00154-s001.zip › ijms-1491862-supplementary.pdf]
